# Breast Cancer in Canadian Women

**DOI:** 10.1186/1472-6874-4-S1-S12

**Published:** 2004-08-25

**Authors:** Heather Bryant

**Affiliations:** 1 Division of Population Health and Information, Alberta Cancer Board, Calgary, Canada, T2N 4N2

## Abstract

**Health issue:**

Although lung cancer is the leading cause of cancer deaths for Canadian women, breast cancer is the most frequently diagnosed. About 5400 women are expected to die from this disease in 2003. In 1998, a woman's lifetime risk of breast cancer was about one in nine.

**Key findings:**

A number of risk factors for breast cancer have been identified. These include advancing age, hormonal factors (eg. early menarche, late menopause and late age at first full-term pregnancy), familial risk, BRCA-1 and BRCA-2 gene mutations, diet and postmenopausal obesity.

Several interventions have been introduced to assist women at high risk for breast cancer, including genetic counseling and testing for women who have strong family histories of breast cancer; selective estrogen receptor modifiers, such as tamoxifen, that has been shown to reduce breast cancer rates; prophylactic mastectomy and screening.

**Data gaps and recommendations:**

Guidelines are unclear in several areas, particularly in screening. Where clinical guidelines are available, health services research or ongoing monitoring (by provincial/territorial cancer agencies) is needed to assess compliance with the guidelines and to ensure equity of access within the provinces/territories.

Key components of organized screening programs need to be established, in part to ensure that screening is carried out in high-quality, co-ordinated programs. There is also a need to develop ways to involve women fully in informed decision-making and to address several policy issues to prevent disparities in access to high-quality services. Patenting issues associated with genetic tests also need to be clarified.

## Background

The last decade has seen breast cancer come to the foreground as one of the chief health concerns of Canadian women, partly because of the importance of breast cancer as a cause of illness and premature mortality, and also as a result of the work of advocacy groups in bringing this issue to public attention. In 1992, a House of Commons Standing Committee report, Breast Cancer: Unanswered Questions, identified a number of issues in breast cancer research, prevention and care. This resulted in a National Forum on Breast Cancer, a major strategic event sponsored by the Canadian Cancer Society, the National Cancer Institute of Canada, the Medical Research Council and Health Canada, held in Montréal in 1993. [1] The legacy of this forum was a coordinated strategy in breast cancer, which has been developed over the past 10 years.

This chapter will provide data on the impact of breast cancer in epidemiologic terms, the progress that has been made in preventing the disease, and the questions – either research- or policy-based – that continue to present themselves to us.

## Methods

This is in large part a review of available literature at the time of publication. Specific data are cited in the Figures, and the methods for their production are listed in the source documents. The work of Health Canada, the National Cancer Institute of Canada and the Alberta Cancer Board is acknowledged in the production of these Figures.

## Results

### Epidemiology in Canada

**Figure 1:**
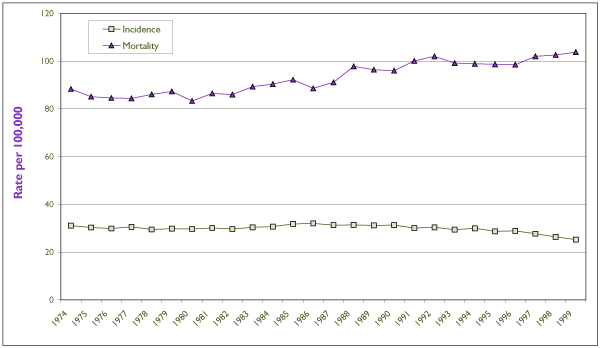
**Age Standardized Incidence and Mortality Rates for Females, Canada, 1974–1999 **Source: National Cancer Institute of Canada. Canadian Cancer Statistics 2003. Toronto, Canada, 2003

Breast cancer is the most common invasive cancer among Canadian women. [2] Age-standardized breast cancer incidence rates increased by 25% among Canadian women between 1973 and 1998, [2] as shown in Figure 1. It appears that much of the increase was in the earlier years of this period. The cause of the increase is not well understood, although some have suggested that changes in reproductive patterns could be partially responsible. [3]

There is also speculation that some of the increase in the late 1980s and early 1990s could have been due to additional detections arising as a result of screening mammography. [4] However, the rates appear to have levelled off since 1993, despite increased use of mammography in the 1990s. [5] Some of this levelling off may have been expected: screening causes an increase in detection in the year of its introduction and, as a result, a decrease in breast cancers found in subsequent years. The degree to which this or other factors may be operating is not well understood and bears further surveillance.

**Figure 2:**
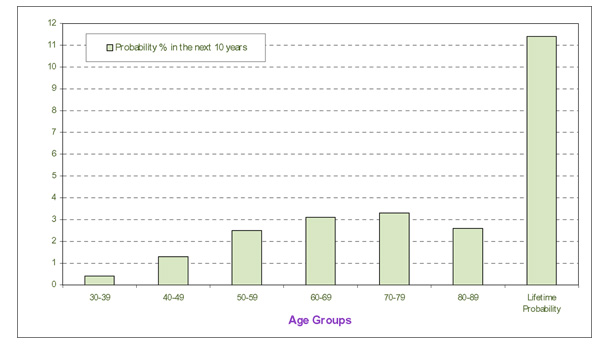
**Probability of Developing Breast Cancer by Age **Source: National Cancer Institute of Canada. Canadian Cancer Statistics 2003. Toronto, Canada, 2003

Most women are aware of the lifetime risk of breast cancer as being close to 1 in 10; in 1998, the risk was about 11.4% or 1 in 9. [2] This is highly age-dependent, however. Risk over the next 10-year period may be a more reasonable number for women and physicians to use to estimate risk, [6] and this increases with age (Figure 2). The risk falls only after the age of 80, probably because other causes of death remove the woman from risk for part of the time.

The news about mortality is somewhat better – the age-standardized mortality rate has fallen by about 15% since 1973, with most of the improvement since 1990. [2] However, breast cancer remains a major contributor to mortality in Canada, and about 5,400 women are expected to die from the disease in 2003. Although breast cancer was the leading cause both of cancer deaths and of potential years of life lost (PYLL) for all causes in the early part of the last decade, [7] it has now been overtaken by lung cancer on both measures. [2] Nevertheless, breast cancer accounts for 94,000 PYLL in Canada, or 6.7% of all premature mortality years for Canadian women.

**Figure 3:**
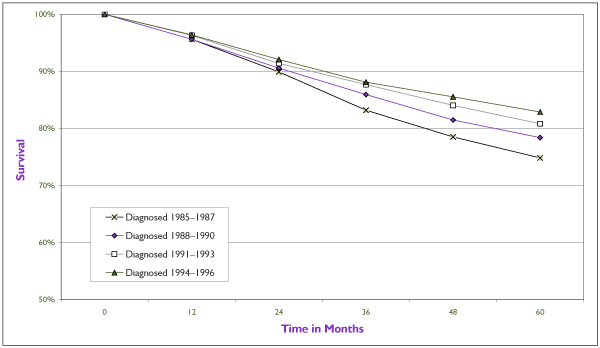
**Breast Cancer Survival Between 1985 and 1996 **Source: Alberta Cancer Board, A Snapshot of Cancer in Alberta 2001. Calgary, Alberta.

The reduction in mortality rates has not resulted from a decrease in the number of cases of breast cancer and must therefore reflect better survival in those affected. There is evidence that this is the case. Data from Alberta indicate temporal trends towards improved 10-year survival [8] (Figure 3). However, even with this evidence it is difficult to know whether the improvements are due to screening (finding cancers at an earlier, more treatable stage) or to better treatment for some or all stages of cancer. [9]

### Provincial/Territorial Variations

Within Canada, the estimated 2002 mortality rates vary from a low of 22 deaths per 100,000 women inSaskatchewan (age-standardized to the 1991 Canadian population) to 29 in Nova Scotia and Newfoundland and Labrador. [2] There are similar variations in age-standardized five-year relative survival rates (1992 diagnosis year), from a low of 76% in Newfoundland and Labrador to a high of 85%in British Columbia. [10] It has been suggested that the latter variations may reflect differences in mammography utilization across the country. [10]

### Ethnic Groups

Little is known in Canada about differences in cancer incidence or mortality across different ethnic or racial groups, as Canadian cancer registries do not collect this information. There is a suggestion that rates are lower in Inuit populations, although there was a trend to an increase in the 1969–1973 and the 1984–1988 periods. [11] Further research and/or enhanced surveillance is needed to determine the impact of cancer on Aboriginal and immigrant populations in Canada.

### International Data

Worldwide, there seems to be some movement towards convergence of breast cancer mortality rates. Rates are higher in North America and northern Europe than in less industrialized and Asian nations. [12] However, rates are declining in industrialized countries such as the United Kingdom, United States, Germany and Canada, and increasing in Japan. These differences seem to have environmental (as opposed to genetic) causes, as migrants from low-risk countries to Canada and Australia tend to acquire higher rates of risk. [13] Recent U.S. analyses point to a decrease in mortality among women born after 1948, although the reasons for this are not well understood. [14]

### Risk Factors for Breast Cancer

The strongest risk factors for female breast cancer – those that raise the individual's risk at least fourfold – include age and country of birth, both described earlier. [15] Familial factors are also important, although to reach this high level of risk an individual would have to have both a mother and a sister with breast cancer. Mutations in the BCRA1 or BCRA2 genes also confer high risk, and these will be discussed later. The other factor to reach this risk level is the presence of atypical epithelial cells in nipple aspirate fluid, although the test that detects the cells is generally used only in research contexts.

### Hormonal Factors

Reproductive and hormonal factors have long been linked to breast cancer risk. The well-known factors include early age at menarche and late age at menopause, or a late age at the first full-term pregnancy. Nulliparity, or never completing a full-term pregnancy, increases the risk of breast cancer after age 40 (the vast majority of breast cancers occur after this age), although pregnancy may confer an increased risk of cancer before the age of 40. [15] These risk factors are all relatively weak on an individual basis, conferring arelative risk of less than double for women with these characteristics compared to women without these characteristics.

Hormone therapy (HT) has been a controversial area in the breast cancer literature. Before publication of the randomized controlled trial known as the Women's Health Initiative (WHI) study in 2002, large pooled analyses of observational studies showed an increase in breast cancer risk among women undergoing HT, which appeared to increase with duration of use. [16] It was thought, however, that this increased risk could be outweighed by potential cardiovascular benefits. The WHI study confirmed an increase in breast cancer risk of about 26% over 5.2 years [17] with combined estrogen/progestin therapy. Although this was a concern, the major finding of the study was the reporting of an increase, rather than the expected decrease, in coronary heart disease. While this may, in part, have been due to the age of the women at the time of study enrolment, it was felt that the overall hazard ratio for the drug was unacceptable, and this portion of the study was discontinued (the estrogen-only trial is still under way). Many organizations are now recommending combined HT only for relief of symptoms at the time of menopause, and Health Canada discourages its long-term use except in limited circumstances. [18]

### Diet and Obesity

Because of the worldwide variations in breast cancer incidence, there have been many studies attempting to link risk to variations in diet. Despite many years of study, there is little conclusive evidence on dietary fat or other putative dietary risk factors.

Post-menopausal obesity increases risk to some degree. If population trends in obesity continue, [19] this may cause a gradual increase in rates in years to come. Physical activity appears to be protective for breast cancer risk, even if activity begins after menopause. [20] Alcohol has been suggested as a risk factor in most studies, [21] and some cohort studies have shown about a 30% increased risk of breast cancer among drinkers. [22,23] However, a moderate use of alcohol is preventive for other diseases, such as diabetes mellitus, so the public health recommendations that should be derived from this are unclear.

### Radiation

Exposure to high levels of ionizing radiation, especially at a young age, is an acknowledged, if somewhat rare, risk factor. [15] The levels known to increase risk are high, and often these types of exposures have already fallen out of favour or have been severely restricted (e.g. use of fluoroscopy in tuberculosis, radiation treatments for acne or thymic enlargement, etc.).

### Familial and Genetic Risk

A family history of breast cancer is perhaps the best-known risk factor. Recent pooling of data from 52 epidemiologic studies indicates that women with no affected first-degree relatives (mother, sister ordaughter) have a 7.8% probability of developing breast cancer by age 80, whereas those with a historyof breast cancer in one first-degree relative have a risk of 13.3%; [24] the risk increases to 21.1% for those with two first-degree relatives. However, less than 1% of women with breast cancer actually have a family history this strong. In fact, eight out of nine women with breast cancer do not have an affected first-degree relative, and the vast majority of those with a family history will not develop breast cancer themselves. [24]

Despite this, there is a small group of women whose familial histories and/or genetic profiles put them at a considerably increased risk. The best-known susceptibility genes, BCRA1 and BCRA2, are believed to have a combined population frequency of about 1.2 per 1,000 women. [25] About 35% of women with a BCRA1 gene defect and 50% of those with a BCRA2 defect would be expected to develop breast cancer by the age of 70. [25] These women also have an increased risk of ovarian cancer, which would be considered in any counselling or surveillance strategies.

### Interventions

#### Genetic Testing

Women who have strong family histories with more than one first-degree relative affected, especially with early-age onset, may be considered for genetic counselling and potentially for familial genetic testing. Women with family histories often overestimate their degree of risk, [26] and counselling helps to put the risks and benefits of such testing in perspective. Women need to understand that not all strong family histories can be linked to single gene defects, and so there is a possibility that such testing will be inconclusive in some families.

Further, the steps to be taken if a genetic defect is found are not entirely clear. Although some would recommend increased mammographic screening, others suggest that women with breast cancer susceptibility genes may actually be more sensitive to radiation, and thus they question the wisdom of this strategy. [27]

At the time of writing, a controversy exists that may limit the availability of genetic testing for Canadian women. [28] Some governments, including several in Europe, are challenging the granting of patents forhuman genes. [29] There will need to be considerably more debate on genetic patenting, both in Canada and worldwide.

#### Selective Estrogen Receptor Modifiers

Some studies are now addressing potential interventional strategies to lower breast cancer risk among women whose current risk is quite high. Tamoxifen is one of a class of drugs known as SERMs, or selective estrogen receptor modifiers. This drug has been shown to reduce breast cancer rates (as well as fractures, as a result of its prevention of osteoporosis) among women whose family history and other risk factors place them at elevated risk. [30] However, there were not enough women with known BCRA1 and BCRA2 defects in this study to make confident conclusions about its use in this group of women. [31] Mathematical models predict that the benefit would be modest, however, with about a 13% to 27% reduction of risk at current estimates. [32]

Unfortunately, tamoxifen has also been associated with increased risk of endometrial cancer and thrombotic (blood clotting) events. This has led to new trials with another SERM, raloxifene. The results of a study designed to look at the effect of raloxifene on osteoporosis prevention showed promising results in breast cancer reduction [33] with little effect on uterine cancer rates. The STAR (Study of Tamoxifen and Raloxifene) trial is now in progress to compare tamoxifen and raloxifene. [34]

#### Mastectomy

The other interventional strategy that could be considered to lower breast cancer incidence in cases of very high risk is prophylactic mastectomy. This has been found to reduce breast cancer risk by about 90%, [35] although occasional cases of breast cancer still occur. Clearly, women need detailed information on the potential risks and benefits of either the surgical or medical preventive strategies.

#### Screening

For well over a decade, Canadian women have been advised that breast screening includes three components: breast self-examination (BSE), clinical breast examination (CBE) and, for some age groups, mammography.

#### Mammography

A report of an international workshop held by the National Cancer Institute (NCI) in 1993 was one of the most influential statements over much of the last decade [36]. This report found that routine mammographic screening for women aged 50 to 69 reduced breast cancer by about a third. However, it noted that for women aged 40 to 49, there was no benefit at 5 to 7 years of screening, and the benefit at 12 years of follow-up, if present at all, was marginal. [36] Another consensus panel, convened in 1997, still found insufficient evidence to recommend routine screening for women in their 40s. [37] Despite this, the NCI chose to make recommendations for routine mammography in this age group. [38] The Canadian Preventive Services Task Force reviewed the issue in 2001 and did not find sufficient evidence to recommend mammographic screening in this age group. [39]

Because women in their 40s must come to some kind of a decision in the face of conflicting recommendations, it is important to provide them with information that will help them make a decision with which they are comfortable. Any screening test involves some risk, as there is always the possibility that a false positive test will result in unnecessary, invasive tests, or that a false negative test will inappropriately reassure a woman when a cancer is present. For women in their 40s, the risk of a false positive test over a decade of biennial screening is estimated to be about 30%, and for 10 screening mammograms in this period about 56%. [40] The sensitivity of mammography is lower before menopause (about 78% versus 90% among women over 50). [41] Thus, there is a risk of having to undergo additional tests because of false positive screens, and there is also about one chance in four that a cancer that is present will not be detected. On the other hand, there may be a small reduction in breast cancer mortality after several years of follow-up. Decision aids that would help women weigh this information and make a personal decision are needed.

For the past year, the debate has taken on another form, however. A reanalysis published in the Lancet in 2001 suggested that mammography was not beneficial in any age group. [42] Because of the controversy generated, the U.S. Preventive Health Services Task Force, the National Cancer Institute and the World Health Organization reviewed their recommendations and, as a result, confirmed the benefits of screening mammography. [43-45]

#### Breast Self-Examination

Another screening procedure, BSE, has also been revisited in the past couple of years. A recent update found that there is no evidence of benefit and some evidence of risk, and so recommended that it not be routinely advised. [46] This again caused controversy, as many women felt that it was the only procedure available to them before the age of routine mammography or between mammography visits. For those women who actively decide to do BSE after a discussion of risks and potential benefits, consideration can still be given to providing high-quality teaching resources so that the procedure is as beneficial as possible.

#### Organized Screening Programs

As early as 1989, there were recommendations in Canada that mammographic screening be done in the context of organized screening programs, [47] a suggestion that was reiterated at the National Forum on Breast Cancer in 1993, [1] when the elements necessary for high-quality screening of the target population were spelled out:

• a population-based outcome goal

• information about the target population

• attention to those hard to reach

• meticulous quality assurance, including equipment and interpretation

• outcome data and analysis

• a woman-centred focus

• information systems and linkages

• coordination with high-quality diagnosis

Between 1988 and 1991, five provinces and one territory inaugurated programs; they are now in place in all 10 provinces and in 2 territories. [48] There has been evidence that provinces and territories that initiated programmatic screening early were more effective in reaching the target population. [49] The programs report on their outcomes nationally and show a high degree of compliance with international standards for cancer detection rates, referral rates and other quality indicators. [48]

One of the quality determinants for screening is a recommended minimum number of readings of3,000 films per radiologist per year, [50] a standard supported by evidence that high-volume readers (more than 5,000 mammograms per year) showed a significantly higher sensitivity than those reading fewer than 3,600 per year. [51] This was achieved without referring more women out for unnecessarytests. Within programs, there is high-volume screening, and evaluation of outcomes is carried out routinely.

**Figure 4:**
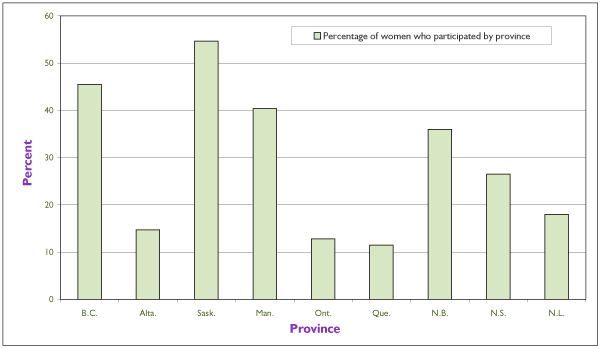
**Proportion of Women Aged 50–69 Who Participated in Provincial Breast Cancer Screening Programs in 1997 and 1998 **Source: Public Works and government Services Canada, 2001. Cat. No. H1-9/13-1998. ISBN 0-662-65807-8

Unfortunately, in most provinces and territories, the majority of women do not receive screening through such organized programs [48] (Figure 4), and much of the screening mammography in the country does not report on quality outcomes. Outside of programs, the minimum number of mammograms required to achieve accreditation is only 480 per year, which is well below the number recommended for high-quality screening. This is a policy issue that will need to be resolved if we are to maximize the gains from screening mammography.

Another measure of quality is the time between screening and diagnosis. Recent studies have shown average delays of about 3.7 weeks from screening to diagnosis in Canada, increasing to 6.9 weeks if a biopsy is involved. [52] Within the programs, goals have been set to minimize this delay.

**Figure 5:**
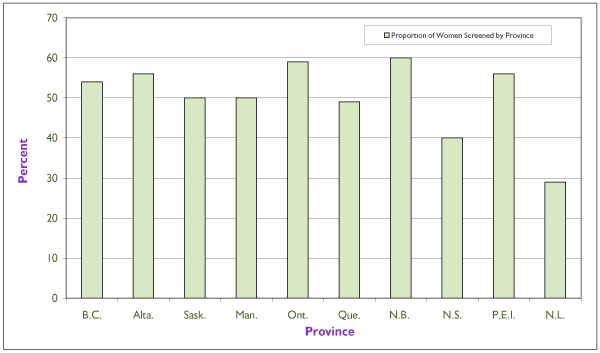
**Proportion of Women Aged 50 to 69 Reporting Having Had a Screening Mammogram Within the Last Two Years, 1996–97 **Source: Statistics Canada. National Population Health Survey, 1996–1997

There are still differences in the ability of organized programs to reach some women for screening. The Canadian population health survey of 1996–1997 showed that only 29% of Newfoundland and Labrador women aged 50 to 69 reported undergoing screening mammography in the previous two years, as compared with 59% in Ontario and 60% in New Brunswick [51] (Figure 5). Mammography use is directly related to education and income, with less mammography being reported by women with less than a high school education and/or lower income. [53]

### Treatment

Regional or provincial variations in treatment practices and access to radiotherapy have been found. [54,55] It is hoped that clinical practice guidelines, developed as part of the national breast screening initiative, [56] will ensure that common treatment standards are in place across the country. There is also a need to ensure that guidelines, once available, are translated into practice.

## Discussion

### Data Limitations

The data presented represent high-quality population-based data available from a number of sources. However, surveillance in Canada could be enhanced by various initiatives. First, there is no readily available data on the staging of cancer across the country, as stage is not routinely collected by cancer registries; this would be valuable in interpreting whether there are differences in time or place in the diagnosis or stage-specific survival of breast cancer. Further, there is no specific information on Aboriginal or other groups, as ethnic origin is not collected on Canadian registries. Finally, while the National Population Health Survey and the Canadian Community Health Survey provide excellent self-report data on screening, enhanced data from actual screening providers in addition to those currently reporting from the screening programs would allow us to get more accurate data on screening prevalence and outcomes across the country.

### Recommendations

#### Policy Implications and Recommendations

There are several areas, particularly in screening, in which the guidelines are unclear and there is a need to develop ways to involve women fully in informed decision making. Several policy issues need to be addressed to prevent disparities in access to high-quality services:

• The patenting issues of genetic tests and thus the availability of testing for women with high-risk histories need to be clarified.

• There should be leadership to ensure that all screening occurs in the context of high-quality, coordinated programs.

• The key components of organized screening programs, identified well over a decade ago, should be put in place.

• When clinical guidelines are known, there is a requirement for health services research or ongoing monitoring by the provincial cancer agencies to assess the adherence levels to guidelines and to ensure equity of access within provinces.

If attention is paid to these issues, we have some hope of minimizing the rates of breast cancer in Canada and further reducing the burden for the over 20,000 women who will develop breast cancer in Canada this year.

## Note

The views expressed in this report do not necessarily represent the views of the Canadian Population Health Initiative, the Canadian Institute for Health Information or Health Canada.
